# Effects of Oat Varieties and Growing Locations on Seed-Borne Fungal Communities

**DOI:** 10.3389/fmicb.2021.724999

**Published:** 2021-12-17

**Authors:** Jianjun Wang, Xuekai Wei, Taixiang Chen, James F. White, Guiqin Zhao, Chunjie Li

**Affiliations:** ^1^State Key Laboratory of Grassland Agro-Ecosystems, Key Laboratory of Grassland Livestock Industry Innovation, Ministry of Agriculture and Rural Affairs, Engineering Research Center of Grassland Industry, Ministry of Education, Gansu Tech Innovation Center of Western China Grassland Industry, College of Pastoral Agriculture Science and Technology, Lanzhou University, Lanzhou, China; ^2^Department of Plant Biology, Rutgers, The State University of New Jersey, New Brunswick, NJ, United States; ^3^Pratacultural College, Gansu Agricultural University, Lanzhou, China

**Keywords:** seed-borne, fungal community, fungal diversity, varieties, locations, oat

## Abstract

Many species of seed-borne fungi are closely allied with seed varieties and growing regions, including many seed-borne pathogens, but their species richness and distribution remain largely unknown. This study was conducted to explore the seed-borne fungal composition, abundance and diversity in *Avena sativa* (B7) and *A. nuda* (B2) seed samples collected from Baicheng (BB), Dingxi (DB) and Haibei (HB) city, using Illumina sequencing techniques. Our results show that a total of 543,707 sequences were obtained and these were assigned to 244 operational taxonomic units (OTUs) with 97% similarity. Oat varieties and growing locations had a significant difference on seed-borne fungal diversity. HB had a higher fungal diversity than BB and DB, Shannon diversity and ACE richness index of fungal in HB seeds was significantly higher than in BB and DB (*P* < 0.05). In different varieties, both taxon richness and evenness of B7 seeds was significantly higher than B2 (*P* < 0.05). A total of 4 fungal phyla and 26 fungal genera were detected. Ascomycota was the dominant phylum and *Alternaria* sp. was the most abundant genus in B2 and B7 oat seeds from different regions. *Mycosphaerella* sp. had a higher abundance in HB7 and DB7, respectively, *Epicoccum* sp. had a higher abundance in HB7 and BB7. The results of alpha and beta diversity analysis revealed the presence of different effects in fungal communities of different varieties and regions of oat, especially in seed pathogenic fungi distribution. Structural equation modeling also explained oat varieties and growing regions have significant influences on seed-borne fungal abundance, composition and diversity. This study demonstrated that the differences of varieties and regions are the main factors resulting in the changes of seed-borne fungal community of oat.

## Introduction

Oat, a cereal of the family Gramineae, is an important source of nutritious food and feed, and widely cultivated in temperate regions of the world, especially in Northern Europe, China, North America, Australia, and Canada (Marshall et al., 2013; Sánchez-Martin et al., 2014). There are two types of cultivated oat in the world, that is covered oat (*A. sativa*) and naked oat (*A. nuda*), and both they are self-pollinated. Covered oat is used for animal feeding due to its high-forage yield, great palatability, high sugar and protein content, lower neutral washing fiber content, while naked oat is used as human consumption because of its cholesterol-lowering properties and high levels of β-glucan, oil, antioxidants, fat-soluble vitamin E and polyunsaturated fatty acids, it has also been recognized as a health food with the benefits which prevents dermatology, hypercholesterolemia, cardiovascular disease and diabetes (Cui et al., 2010; Sterna et al., 2014; Guo et al., 2017; Munkhtuya, 2017; Sadras et al., 2019). They are all grown for producing grain, green pasture and hay to provide an excellent source of emergency forage before winter and health food. In addition, oat also has other characteristics, such as drought-resistance, barren-tolerance, strong adaptability and stable yield (Xu, 2005). Based on its excellent characteristics, oat has a significant importance for human health and livestock nutrition. In recent years, with the increase in demand and use for oats and their products, oat has attracted considerable interest for its high nutritional value and has become a high efficiency crop of degraded grasslands worldwide, oat research has also become the focus of food science and animal husbandry development (Guo et al., 2012; Marshall et al., 2013).

Seeds are the basic input for the production of many plants and known to carry many microorganisms. Protein and starch rich cereal crops are also highly susceptible to various diseases that can cause considerable losses to yield (Habib et al., 2011). Oat seeds are often stored for human foods, animal feeds or seeds. Seed-borne microorganisms play a vital role in affecting the health and quality of oat seeds during storage; they are responsible for both pre- and post-emergence death of oats, affecting seedling nutrition and vigor, and thus cause reduction in seed germination and seedling growth (Butt et al., 2011; Hajihasani et al., 2012). Fungi are important factors reducing seed viability during seeds storage. Seed and seedling diseases are caused by seed-borne fungi that can survive in infected seeds (Aslam et al., 2015). Seed-borne diseases caused by fungi are relatively difficult to control when the fungal hyphae get established in seeds and become dormant during storage (Attiq-ur-Rahman et al., 2018). These seed-borne pathogens are present externally or internally and may grow on stored oat seeds. They have direct or indirect influences on seed production and food security of oat, because they can cause seed abortion, rot and necrosis, reduction of germination capacity as well as seedling damage (Habib et al., 2011; Rahmatzai and Saifulla, 2011; Singh et al., 2011; Jelena et al., 2012). Seeds are major transmission contributors of pathogenic fungi, diseases can be spread through seeds from one region to another to result in the development of disease at later stages of plant growth by systemic or local infections (Ahmed et al., 2017). Although damage caused by seed-borne pathogens is not always recognized, seed treatment before sowing may improve seed germination capacity. Pathogen free seeds are a precondition for optimal seed germination and seedling growth. It is necessary to study seed-borne fungi and to reduce the pathogen carrier rate of oat seeds and increase the production and quality of feeds and seeds.

Numerous previous studies have reported the isolation and identification of seed-borne fungi in different seeds, especially in cereal and grass seeds. Several seed-borne pathogens have been reported worldwide, including species belonging to genera of *Alternaria*, *Fusarium*, *Aspergillus*, *Penicillium* (Li and Nan, 2000; Zhang, 2005; Chen, 2010; Jelena et al., 2012; Pathak and Zaidi, 2013; Aslam et al., 2015; Chen and Nan, 2015). Furthermore, the above fungi were also isolated from oat seeds of different varieties and regions (Jing et al., 2012; Nie et al., 2019). However, these studies were limited in fungal detection and types by culture-based approaches and morphological characteristics. There are no reports regarding the structure, diversity and relative abundance of fungal communities in oat seeds. The influences of oat varieties and growing regions on fungal diversity of oat is also poorly understood. It is difficult to predict the production of mycotoxins because several fungal species are difficult to grow on media, and many are slow growing. Thus, in order to comprehensively understand the communities of seed-borne fungi in oat seeds, the present study was carried out to examine the distribution and diversity of seed-borne fungi in naked and covered oat seeds collected from three different locations of China by using MiSeq sequencing.

The aim of this study was to characterize the main fungal species associated with naked and covered oat seeds from different locations. The specific objectives in the present study were (1) to investigate the structure diversity and relative abundance of fungi in oat seeds using MiSeq sequencing, and (2) to directly compare the influence of different oat varieties and growing regions on fungal communities. Based on the previous findings, we also hypothesized that (1) different oat varieties and growing regions could differ in their seed-borne fungal community structure, and (2) the distribution of pathogenic fungi in oat seeds may be related to oat varieties and locations.

## Materials and Methods

### Collection of Seed Samples

This paper focused on two common oat cultivars: “Baiyan 2” (B2) and “Baiyan 7” (B7) bred by Baicheng Academy of Agricultural Sciences, Jilin Province, China. They were all planted in three different provinces of northern China for several years. The seeds of *A. sativa* (B7) and *A. nuda* (B2) were, respectively, obtained from one plant of three provinces in 2017. They all were separately planted in three provinces, and the ripe seeds of B2 and B7 were also harvested from three provinces at full-ripe stage in 2018: (1) Baicheng City, Jilin Province (45°37′ N, 122°48′ E, Altitude 155 m), seeds were marked as BB-2 and BB-7. (2) Dingxi City, Gansu Province (35°56′ N, 104°60′ E, Altitude 1,883 m), DB-2 and DB-7. (3) Haibei City, Qinghai Province (36°56′ N, 100°56′ E, Altitude 3,012 m), HB-2 and HB-7. Mean annual temperature (MAT) and precipitation (MAP) data of the three sites in 2018 were gained from local weather stations and presented in Table 1. The harvested seeds of oat were healthy-looking without any visual symptoms of any disease, and divided into six different groups based on their collected site and covered or naked characteristics (BB2, BB7, DB2, DB7, HB2, and HB7). Each group contain 250 seeds and a total of 1,500 B2 and B7 seeds from three areas were randomly selected and used in present study. All seeds were placed in sterilized polythene bags and stored at a constant 4°C for 5 months prior to analysis.

**TABLE 1:** Meteorological information and soil properties data for three different locations of oat seed production in 2018.

Sites	MAT (°C)	MAP (mm)	PH	TN (g/kg)	TP (g/kg)	AK (g/kg)
Baicheng	4.9	407.9	8.2	1.09	0.67	0.11
Dingxi	6.4	415.2	8.14	0.83	0.81	0.12
Haibei	−0.5	366.4	8..21	1.28	0.72	0.25

### Soil Physical and Chemical Properties Analysis

Soil samples were collected from three different oat growing sites where seeds were obtained. Within each growing site, three quadrats were randomly selected near to the plant, and three 0–10 cm soil samples were, respectively, collected per quadrat using a 20 cm soil auger. All soil samples were air-dried, passed through a 2-mm sieve, and stored at 4°C prior to being analyzed for the physical and chemical properties. Soil pH was analyzed using a Star A211 pH meter (Thermo Orion, Beverly, MA, United States) at a ratio of 1:5 (soil to water, w/w) (Little, 1992). Available potassium (AK) was extracted and analyzed with ammonium acetate and flame photometry (Helmke and Sparks, 1996). Total nitrogen (TN) and total phosphorus (TP) were determined using a continuous flow analyzer (FIAstar 5000 Analyzer) (Zhao et al., 2014). The data on soil properties were listed in Table 1.

### DNA Extraction and Polymerase Chain Reaction (PCR) Amplification

A 50 seeds of each group was a treatment and each treatment had five replicates in this experiment. Seed samples from six groups were surface sterilized with 1% NaOCl solution for 2 min followed by five washings with sterilized water. After surface sterilization seeds were blotted dry and used to extract total DNA of oat seeds. Extraction from seed samples was done using a Plant Genomic DNA Kit (Omega Bio-Tek, Norcross, GA, United States) according to the manufacturer’s protocol. DNA was diluted and used as a template for polymerase chain reaction (PCR) amplification with specific barcoded primers by KAPA High-Fidelity Hotstart Ready Mix PCR Kit (KAPA-Bio, Woburn, MA, United States). The fungal ITS1 region was amplified using the specific primer pair of 2045F (GCATCGATGAAGAACGCAGC) and 2390R (TCCTCCGCTTATTGATATGC) (Lei, 2017). PCR was conducted in a 25 μL reaction mixture consisting of 1 μL of extracted DNA, 1 μL of each 5 μM primer and KAPA High-Fidelity Hotstart Ready Mix (KAPA-Bio, Woburn, MA, United States). The PCR conditions were set as follows: denaturing at 95°C for 5 min followed by 25 cycles (94°C for 30 s, 55°C for 30 s and 72°C for 30 s), and a final extension at 72°C for 10 min. The expected size of amplified products were confirmed by 1.5% agarose gel stained with GoldView and the PCR products were purified with QIAquick Gel Extraction Kit (Qiagen Sciences, United States). Finally, amplicons were submitted to Biomarker Technologies Co., Ltd (Beijing, China) for sequencing on the Illumina HiSeq 2500 platform.

### Bioinformatic Analyses

The sequencing reads were assembled and filtered, sequences with a quality score ≥20 were assigned to different operational taxonomic units (OTUs) at 97% sequence similarity by using QIIME software (Version 1.7.0). Among the sequences of five replicates in each treatment, three reps sequences with higher quality score (>20) and higher sequence similarity (>97%) were selected for further analysis. According to the principle of the algorithm, low frequency and singleton OTUs were eliminated, and the representative sequences of OTUs were selected and classified to construct OTU abundance tables and annotate fungal species based on USEARCH v11 software and UNITE database^1^ (Edgar, 2010; Kõljalg et al., 2013). OTUs at genus and species levels were mainly used in this study. Subsequent analysis of alpha and beta diversity is based on this output of standardized data.

### Alpha and Beta Diversity Analysis

The diversity of the samples was studied through alpha diversity and beta diversity using the OTUs. Diversity indexes were calculated and displayed using Mothur (version v.1.30) and R software (Version 3.6.1). Community richness was determined with the Chao1 and Ace indices, while community diversity was determined with the Simpson and Shannon indexes. The indexes of Chao1, Ace, Simpson and Shannon were calculated by the following formula:

Chao1 index (Chao and Bunge, 2002): Chao1 = *S*_*obs*_ + $\frac{F_{1}\left(F_{1}-1\right)}{2\left(F_{2}+1\right)}$

Where *S*_*obs*_ represents the number of observed OTUs, *F*_1_ and *F*_2_ are the number of singletons and doubletons in each sample.

Ace index (Pfeiffer et al., 2013): *S*_*ace*_ = *S*_*abund*_ + $\frac{S_{rare}}{C_{ace}}+\frac{F_{1}}{C_{ace}}\gamma_{ace}^{2}$

Where *S*_*abund*_ represents the number of more than abundant OTUs, *S*_*rare*_ is the number of less than or equal to abundant OTUs, *F*_1_ is the number of singletons in each sample, and ${}_{ace}^{2}$ is the estimated coefficient of variation for rare OTUs.

Simpson index (Simpson, 1943): $D_{s}=1-\sum_{i=1}^{s}P_{i}^{2}$

Shannon index (Shannon, 1949): $H^{\prime }=-\sum_{i=1}^{s}\left(P_{i}log_{2}P_{i}\right)$

Where *s* is the number of OTUs, *Pi* is the proportion of the fungal community represented by the OTUs.

For beta diversity analysis, principal coordinates analysis (PCoA) of seed-borne fungal communities were performed using R software by pairwise Bray–Curtis dissimilarity based on OTU level. Redundancy analysis (RDA) among seed-borne fungal community composition and meteorological information and soil properties was performed by CANOCO for Windows 4.5.

### Statistical Analysis

The data analyses and figure generations were performed based on the group of BB2, BB7, DB2, DB7, HB2, and HB7. Data analyses were performed with SPSS 22.0 (SPSS, Inc., Chicago, IL, United States). Differences in seed-borne fungal community composition and alpha diversity under different varieties and regions were tested using two-way analysis of variance (Two-way ANOVA). A repeated-measures ANOVA with Fishers Least Significant Differences (LSD) test was used to determine whether differences between means were statistically significant. Statistical significance was defined at the 95% confidence level. A network analysis was performed with a CoNet Cytoscape plug-in (König and Holzhütter, 2010), to explore the linkage among different fungal taxa based on 97% OTU identity. The ecological guild of the fungal OTUs was parsed using Funguild tools, the species information of seed-borne fungi was annotated into three types according to their nutritional patterns: pathotroph, symbiotroph and saprotroph using species with highest confidence support (Nguyen et al., 2015).

Structural equation modeling (SEM) was used to describe the potential causal relationships between explanatory variables and seed-borne fungal diversity. SEM analysis was performed with IBM SPSS Amos 24.0 (Amos Development Co., Greene, ME, United States). The strength of direct and indirect relationships in different variables and areas was calculated based on the results of linear regression. The SEM models based on the potential relationships and known factors among the drivers of seed-borne fungal diversity were constructed, and a step-wise fitting procedure was used to achieve best-supported model based on GFI (Goodness of Fit Index), SRMR (Standardized Root Mean square Residual) and RMSEA (Root Mean Square Error of Approximation).

## Results

### Diversity of Seed-Borne Fungal Community

**FIGURE 1:**
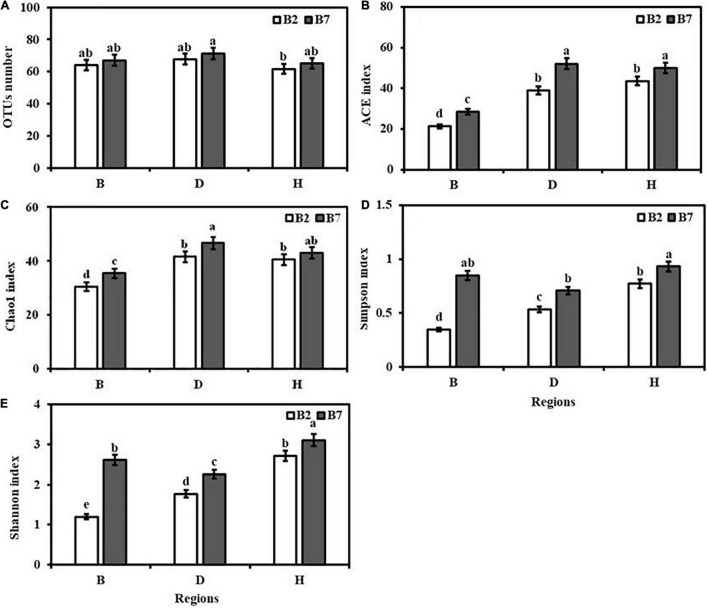
Diversity indices of seed-borne fungal communities of different varieties and regions of oat. **(A)** OTU number. **(B)** ACE index. **(C)** Chao1 index. **(D)** Simpson index. **(E)** Shannon index. Values are means ± standard error (SE), and bars indicate SE. Different lowercase letters stand for significant differences at 0.05 level.

Analysis showed the presence of variable levels of seed-borne fungal diversity in different varieties and areas of oat. Varieties and growing locations had significant effects on the diversity and richness of seed-borne fungal communities. No significant differences were observed in OTU numbers of different varieties and areas of oat seeds (Figure 1A). The Chao 1 and ACE indexes of BB2 and BB7 were significantly lower than other oat seeds (*P* < 0.05), which indicated that BB2 and BB7 had lower seed-borne fungal community richness than other oat seeds (Figures 1B,C). The Shannon and Simpson indices of BB2 were significantly lower than DB2 and HB2, while DB7 were significantly lower than BB7 and HB7 (*P* < 0.05), which showed BB2 and DB7 had lower seed-borne fungal community diversity than other oat seeds (Figures 1D,E). For different oat varieties, B7 had higher seed-borne fungal community richness and diversity than B2 (Figure 1).

Varieties (V) had significant effects on Shannon index and Simpson index, while regions (R) had significant effects on the index of Chao1, Shannon and Simpson (*P* < 0.05). However, the interaction of V × R for OTUs number, Chao1 index and Shannon index was not significant, the index of ACE and Simpson was obvious (Table 2).

**TABLE 2:** Results of two way ANOVA for the effects of varieties (V) and regions (R) on diversity indexes of seed-borne fungal communities in oat.

Treatment	df	OTUs number		ACE index		Chao1 index		Simpson index		Shannon index	
		*F*-value	*P*	*F*-value	*P*	*F*-value	*P*	*F*-value	*P*	*F*-value	*P*
V	1	1.815	0.184	2.982	0.056	2.756	0.197	6.609	<0.001	8.227	<0.001
R	2	4.339	0.033	1.554	0.244	4.73	<0.001	8.882	<0.001	4.34	<0.001
V × R	2	0.813	0.563	1.898	<0.001	1.118	0.133	2.445	<0.001	1.042	0.437

**FIGURE 2:**
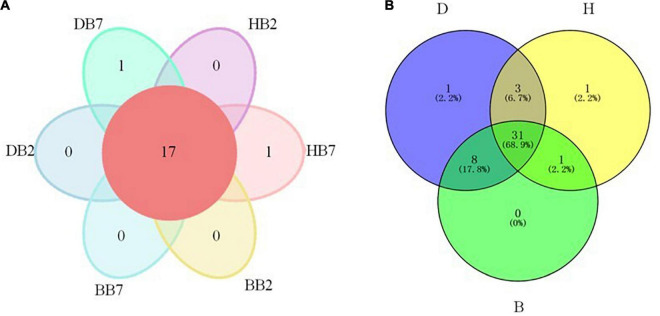
OTU analysis of oat seed samples of different varieties **(A)** and regions **(B)**. OTU of not unique in a single sample or not common among all samples is not shown in the petal diagram **(A)**.

Venn diagram analysis demonstrated 17 OTUs were found in all six groups. For different groups, DB7 and HB7 groups all contained a unique OTU, while DB2, HB2, BB2, and BB7 did not have unique OTUs, respectively (Figure 2A). As to the groups of three different regions, 31 OTUs were found in all three groups. DB and HB groups contained 1 unique OTU, respectively. The significant overlap and the largest number (8) of OTUs were shown between DB and BB groups. In contrast, the HB group shared only 1 and 3 OTUs with the BB and DB groups, respectively (Figure 2B).

### Composition of Seed-Borne Fungal Community

**FIGURE 3:**
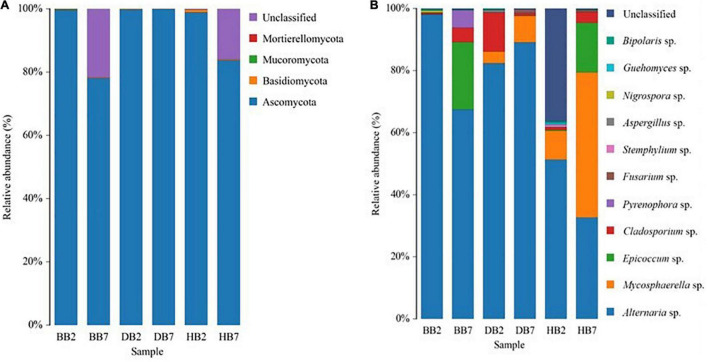
Relative abundance of dominant fungal phyla **(A)** and genera **(B)** in different oat seed samples.

The fungal community compositions were analyzed according to their relative abundance at the taxonomic phylum and genus level, separately. Ascomycota was the most dominant phylum in all seed-borne fungal communities in different oat seeds. It accounted for 77 to over 99% of the fungal sequences in the samples. Basidiomycota was mainly presented in HB2 and HB7, and it was the second dominant phylum after Ascomycota (Figure 3A). *Alternaria* sp. was the most abundant genus of seed-borne fungi in all oat seeds, it accounted for 33 to over 97% of the fungal reads in all samples. The following ten most abundant genera in oat seeds were *Mycosphaerella* sp., *Epicoccum* sp., *Cladosporium* sp., *Pyrenophora* sp., *Fusarium* sp., *Bipolaris* sp., *Guehomyces* sp., *Aspergillus* sp., *Stemphylium* sp., and *Nigrospora* sp. The genus *Mycosphaerella* was the common genus in HB2, HB7, DB2, and DB7, whereas *Epicoccum* sp. was also one of the dominant genera in BB7 and HB7, and *Fusarium* sp. was mainly presented in DB7 and HB7 (Figure 3B).

**FIGURE 4:**
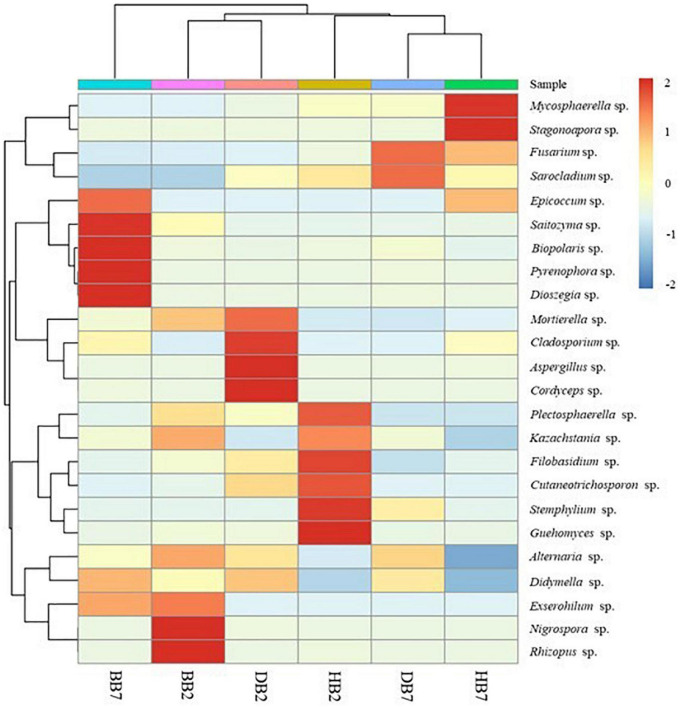
Cluster analysis heatmap of oat seed samples and genus level species abundance.

The distribution and abundance of species at genus level was plotted using heatmap cluster analysis where each row corresponded to a fungal genus and each column corresponded to the oat sample. The vertical clustering showed the similarity of the abundance of different species among the oat samples, and the horizontal clustering represented the similarity of species richness in different samples. The higher relative abundance of fungal species in oat samples, the more intense red color at the corresponding position in the heatmap, blue was the opposite (Figure 4). The results showed the fungal abundance of BB7 was significantly different from other oat seeds. Otherwise, there was a higher fungal abundance similarity between HB7 and DB7; high similarity also existed in BB2 and DB2. The genera *Mycosphaerella*, *Stagonospora*, *Fusarium*, *Sarocladium*, *Epicoccum*, *Saitozyma*, *Bipolaris*, *Pyrenophora*, and *Dioszegia* had a higher abundance in B2. High abundance genera found in B7 were *Mortierella*, *Cladosporium*, *Aspergillus*, *Cordyceps*, *Nigrospora*, *Rhizopus*, *Plectosphaerella*, *Kazachstania*, *Filobasidium*, *Cutaneotrichosporon*, *Stemphylium*, and *Guehomyces*.

### Relationship Between Seed-Borne Fungi and Growing Regions of Oat Seeds

**FIGURE 5:**
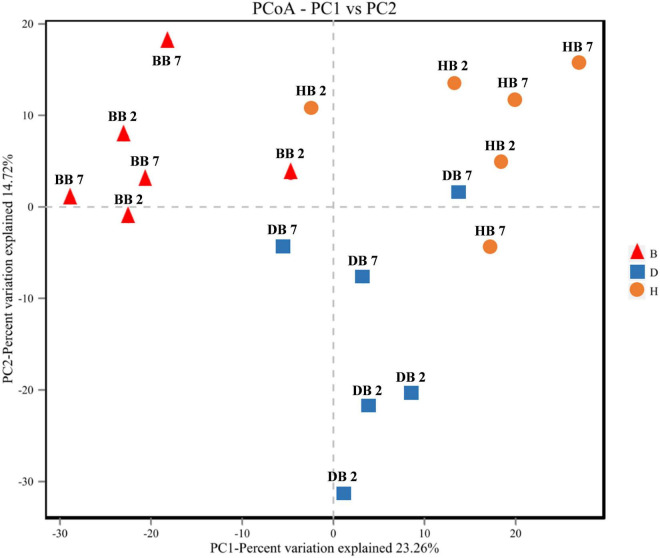
Principal coordinates analysis (PCoA) of fungal diversity in oat seed samples from three region samples.

Principal coordinates analysis of seed-borne fungal diversity based on the OTU level was performed using Bray-Curtis dissimilarities. The results of PCoA indicated that the composition of the seed-borne fungal community was significantly different among three different growing locations, and the fungal community of oat seed samples in the same regions had a higher similarity (Figure 5).

**FIGURE 6:**
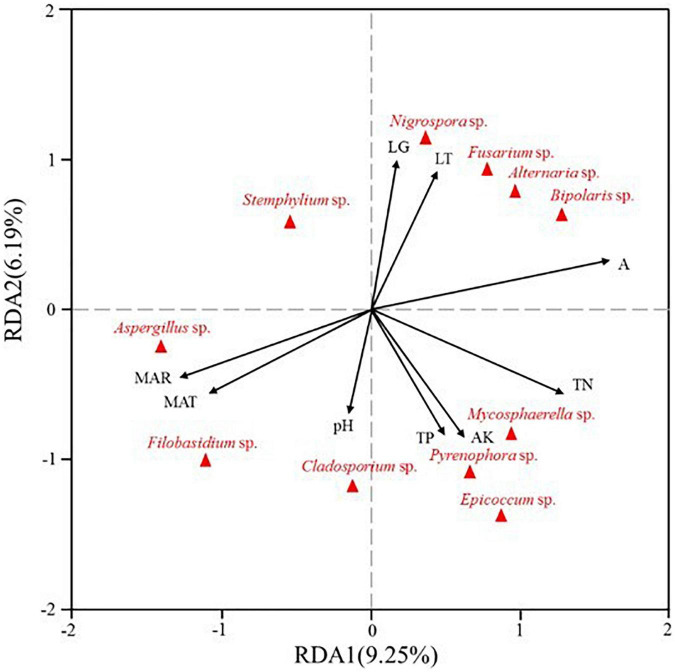
Redundancy analysis (RDA) of relative abundance of seed-borne fungal genera from three different regions (LG: longitude, LT: latitude, A: altitude, MAR: mean annual rainfall, MAT: mean annual temperature, TN: total N, TP: total P, AK: available K, pH).

Redundancy analysis was also used to investigate the influences of meteorological information and soil properties on seed-borne fungal community assemblages. The first and second axis of RDA explained 9.25 and 6.19% of the variance, respectively. For the fungal communities, longitude (LG), latitude (LT) and altitude (A) were closely positively related to the abundance of *Nigrospora*, *Fusarium*, *Alternaria*, and *Bipolaris*. Furthermore, the abundance of *Mycosphaerella*, *Pyrenophora*, *Epicoccum*, and *Cladosporium* were positively related to total N (TN), total P (TP), available K (AK) and pH, while *Aspergillus* and *Filobasidium* abundance was positively related to mean annual rainfall (MAR) and mean annual temperature (MAT) (Figure 6).

### FUNGuild and Network Analyses of Seed-Borne Fungal Communities

**FIGURE 7:**
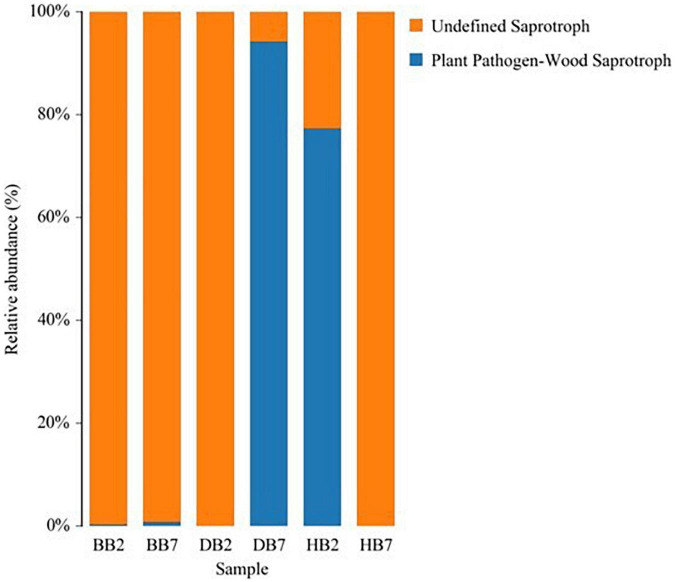
Fungi Functional Guild (FUNGuild) of relative abundance in different oat seed samples.

The application of FUNGuild to three high-throughput sequencing datasets of different trophic modes (pathotroph, symbiotroph, and saprotroph) was analyzed by using a database and an accompanying bioinformatics script. Across these three datasets, FUNGuild detected all three main trophic modes and 12 guilds, and the fungal species with highest confidence were selected. The largest group of guilds in DB7 and HB2 was plant pathogens and wood saprotrophs, and they had a higher guild abundance in DB7 and HB2. With regard to sequence richness, the undefined saprotrophs dominated the BB2, BB7, DB2, and HB7 datasets. Ascomycota was a major phylum of pathotroph and Basidiomycota was a main phylum of saprotroph (Figure 7).

**FIGURE 8:**
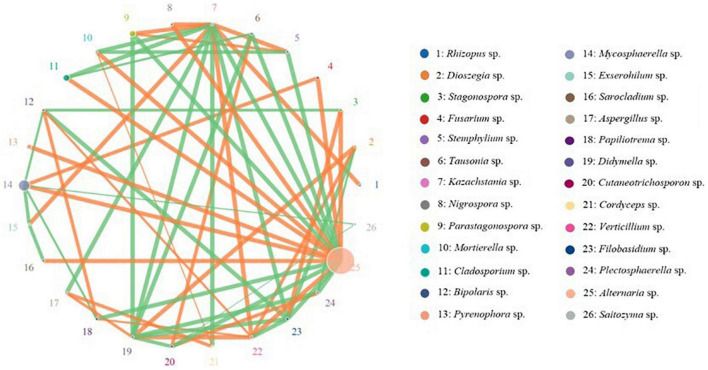
Network analysis on the fungal communities in different oat seed samples. Node represents species and its size represents abundance. The line represents correlation between two species, line thickness represents strength of correlation. The orange line represents a positive correlation, whereas the green line represents a negative correlation.

The OTU network analysis was used to explore the relationships among different fungal taxa. The abundance and relativity of each species in each sample was analyzed by network analysis, and the 26 fungal genera with the highest correlation were shown in the network figure. The results showed the network of 26 fungal genera had 59 significant correlations (edges), and more than half of the nodes were positively correlated, especially in the genera *Alternaria*, *Fusarium*, *Rhizopus*, *Stagonospora*, *Pyrenophora*, *Aspergillus*, and *Verticillium*. In addition, the interaction among fungal taxa was extremely intimate in oat seeds (Figure 8).

### Influencing Factors of Fungal Community Diversity

**FIGURE 9:**
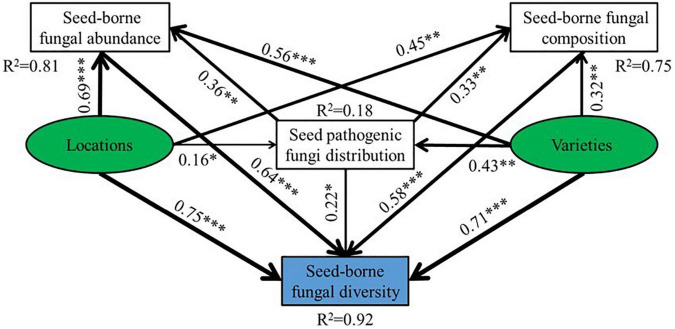
Structural equation model (SEM) showed the effects of habitat and varieties on seed pathogenic fungi distribution, seed-borne fungal abundance, composition and diversity of oat. The arrows reflect the causal relationships, numbers close to lines are standardized path coefficients, the thickness of the black (positive) and red (negative) paths indicates the strength of the relationships, *P < 0.05, ^**^P < 0.01, ^***^P < 0.001 (CFI = 0.994, RMSEA = 0.053, SRMR = 0.039).

The structural equation model (SEM) was established to examine the direct and indirect effects of oat seed varieties and collection locations on seed-borne fungal community diversity. Varieties and regions affected the seed-borne fungal diversity by affecting seed-borne fungi abundance, seed pathogenic fungi distribution and seed-borne fungi composition. Furthermore, the varieties and regions had positive effects on seed-borne fungi abundance, seed pathogenic fungi distribution, seed-borne fungi composition and diversity. The SEM explained 81, 18, 75, and 92% of the variation in seed-borne fungi abundance, seed pathogenic fungi distribution, seed-borne fungi composition and diversity (Figure 9).

## Discussion

Fungi are significant in oat seeds and function in pathogenesis, symbiosis and decomposition, and they also can promote seed germination and nutrient absorption (Han et al., 2016). In recent years, seed-borne fungal diversity in oat seeds have been traditionally detected and classified through morphological features and molecular sequencing technology (Jing et al., 2012; Nie et al., 2019). However, there is little known about the effects of varieties and growing locations on the non-cultured seed-borne fungal community diversity and composition in oat seeds. Therefore, the present study was initiated to determine the effects of different varieties and regions on seed-borne fungal abundance, composition and diversity of oat seeds by using Miseq high-throughput sequencing. The results indicated that fungal abundance, composition, diversity and pathogenic fungi distribution of oat seeds were significantly affected by oat varieties and growing regions.

### Seed-Borne Fungal Community Composition

Previous studies have been carried out to analyze the seed-borne fungal community composition of many different plant seeds. In our study, Ascomycota was a dominant and widespread phylum in all seed-borne fungal communities of different oat seeds. Our results demonstrated the earlier findings on *Phragmites australis* (Ernst et al., 2003), *Elymus nutans* (Guo et al., 2020), and *Phragmites australis* (Kalu et al., 2021), which showed this phylum as a common and dominant phylum. A total of 10 most abundant genera in different oat seeds were *Alternaria*, *Mycosphaerella*, *Epicoccum*, *Cladosporium*, *Pyrenophora*, *Fusarium*, *Bipolaris*, *Guehomyces*, *Aspergillus*, *Stemphylium*, and *Nigrospora*, respectively. These fungal genera had a high incidence in oat seeds, especially the genus of *Alternaria*. It is noteworthy to mention that some of these genera were also observed in seeds of other plants, such as *Medicago* (Li and Nan, 2000), *Phaseolus vulgaris* (Domijan et al., 2005), *Lolium perenne* (Zhang, 2005), *Onobrychis viciifolia* (Chen, 2010), and *Elymus sibiricus* (Chen and Nan, 2015), etc. Furthermore, Al-Bulushi et al. (2017), Lei (2017), and Gao (2018) also found that the genus of *Alternaria* was the dominant fungal species in *Phoenix dactylifera*, *Trifolium repens*, and *E. nutans* during storage by Illumina MiSeq sequencing analysis. Thus, these fungal genera are major components of the seed-borne fungal community.

### Seed-Borne Fungal Community Diversity

The seed-borne fungal community diversity of different oat samples was studied using α-diversity and β-diversity using the OTUs. The α-diversity indices of fungal communities of *A. sativa* and *A. nuda* oat seed samples obtained from three different locations had significant differences, illustrating that the richness and evenness of seed-borne fungal community species were significantly affected by oat varieties and growing locations. Moreover, the β-diversity results indicated growing locations had significant effects on the composition and diversity of seed-borne fungal communities. These results are somewhat similar to the study in *L. perenne* and *T. repens*, which explained the seed-borne fungal community of seeds from different regions was quite different (Lei, 2017). Similar results have also been found in other studies. Nie et al. (2019) found that the composition of seed-borne fungal communities of oat collected from two different regions showed obvious differences. Meanwhile, Chen (2010) and Cao et al. (2019) had similar results in the study of *O. viciifolia* and *Zea mays*; they demonstrated different seed varieties had different influences on the seed-borne fungal community. These results of α-diversity and β-diversity demonstrate a strong varietal and environmental influence on host plant selection on seed-borne fungal species, and they are all closely related to the seed-borne fungal community diversity.

### Relationships Among Seed-Borne Fungi, Seed Varieties and Environmental Factors

Varieties and growing locations can not only affect the seed germination and seedling growth of plants, but they can also influence the diversity and composition of fungal communities associated with plant seeds (Vardhini and Rao, 2003; Chen, 2010; Jelena et al., 2012; Mamun et al., 2018). Previous studies indicated that the diversity and composition of seed-borne fungal communities was affected by some biotic and abiotic factors, such as seed varieties, fertilization, altitude and temperature, etc. (Pathak and Zaidi, 2013; Guo et al., 2017; Gao, 2018). These factors are closely related to the composition and diversity of seed-borne fungi. Our results have suggested that varieties had significant effects on Shannon and Simpson index, while growing regions had significant effects on the index of Chao1, Shannon and Simpson, their interaction had obvious influences on the index of ACE and Simpson. At the same time, HB had a higher fungal richness than DB and BB. The variety B7 was also higher in fungal richness than B2. The SEM also revealed varieties and habitats had positive effects on seed-borne fungal abundance, composition and seed pathogenic fungi distribution. Thus, the differences of Chao1, ACE, Shannon and Simpson illustrated that the diversity and composition of fungal communities in oat seeds are largely determined by the seed varieties and growing locations.

### The Interaction Patterns of Seed-Borne Fungal Genera in Oat

It has long been noted that seed-borne fungi play an important role in plant growth and survival, especially in seeds storage and germination (Hajihasani et al., 2012). With future study on seed-borne fungi, the positive or negative effects of fungi species on host seeds and seedling growth may be elucidated. Some seed-borne fungal species can promote seed germination, like *Fusarium* sp. and *Penicillium* sp. (Tamura et al., 2008). On the other hand, many fungal species are pathogenic and harmful to many host plants (Jelena et al., 2012; Guo et al., 2017). Therefore, these fungal species have positive and negative influences on host seeds. In nature, microbial communities are established in specific niches through mutualistic (positive), competitive (negative) and commensalistic (neutral) networks among different microbial groups (Faust and Raes, 2012; Guo et al., 2020). In this study, the interaction patterns of seed-borne fungal genera in oat were also analyzed by using network analysis. We found a greater number of positive fungus-fungus associations than negative associations, this phenomenon could be seen in genera *Alternaria*, *Fusarium*, *Rhizopus*, *Stagonospora*, *Pyrenophora*, *Aspergillus*, and *Verticillium*. *Alternaria* sp., not only has a higher abundance, but it also has more associations with other fungal genera. The fungal community network in oat seeds is controlled by a large number of taxa, which is also closely interconnected to have effects on the overall fungal community. These fungi also play key roles in oat seed survival and establishment of the next generation’s seed-borne fungal community (Han et al., 2016; Guo et al., 2020).

## Conclusion

The results of this work demonstrated the diversity and richness of seed-borne fungi vary with oat varieties and growing regions. *Alternaria* species was the most abundant genus in oat seeds. Additionally, the seed-borne fungal community of oat seeds was dominated by the fungal genera *Mycosphaerella*, *Epicoccum*, *Cladosporium*, *Pyrenophora*, *Fusarium*, *Bipolaris*, *Guehomyces*, *Aspergillus*, *Stemphylium*, and *Nigrospora*. The differences of varieties and environments are the main factors resulting in the changes of seed-borne fungal community of oat. Additionally, the positive and negative associations in the fungal community existed in oat seeds; these associations may play roles in oat seed survival and establishment of the next generation’s seed-borne fungal community. Further research is needed to explore the ecological function of seed-borne fungi, and the effects on seed vigor, and nutritional quality of oat during storage.

## Data Availability Statement

The datasets presented in this study can be found in online repositories. The names of the repository/repositories and accession number(s) can be found in the article/supplementary material.

## Author Contributions

CL conceived and designed the experiments. JWa performed the experiments and wrote the manuscript. XW and TC performed the experiments. GZ and JWh revised the manuscript. All authors contributed to the article and approved the submitted version.

## Conflict of Interest

The authors declare that the research was conducted in the absence of any commercial or financial relationships that could be construed as a potential conflict of interest.

## Publisher’s Note

All claims expressed in this article are solely those of the authors and do not necessarily represent those of their affiliated organizations, or those of the publisher, the editors and the reviewers. Any product that may be evaluated in this article, or claim that may be made by its manufacturer, is not guaranteed or endorsed by the publisher.

## Footnotes

1
https://unite.ut.ee
